# Active Fantasy Sports: Rationale and Feasibility of Leveraging Online Fantasy Sports to Promote Physical Activity

**DOI:** 10.2196/games.3691

**Published:** 2014-11-25

**Authors:** Arlen C Moller, Sara Majewski, Melanie Standish, Pooja Agarwal, Aleksandra Podowski, Rebecca Carson, Biruk Eyesus, Aakash Shah, Kristin L Schneider

**Affiliations:** ^1^Department of PsychologyIllinois Institute of TechnologyChicago, ILUnited States; ^2^Department of Preventive MedicineNorthwestern UniversityChicago, ILUnited States; ^3^Department of PsychologyRosalind Franklin University of Medicine and ScienceNorth Chicago, ILUnited States

**Keywords:** physical activity, games for health, active video game, exergame, asynchronous, social support, multiplayer, enjoyment, intrinsic motivation, sports

## Abstract

**Background:**

The popularity of active video games (AVGs) has skyrocketed over the last decade. However, research suggests that the most popular AVGs, which rely on synchronous integration between players’ activity and game features, fail to promote physical activity outside of the game or for extended periods of engagement. This limitation has led researchers to consider AVGs that involve asynchronous integration of players’ ongoing physical activity with game features. Rather than build an AVG de novo, we selected an established sedentary video game uniquely well suited for the incorporation of asynchronous activity: online fantasy sports.

**Objective:**

The primary aim of this study was to explore the feasibility of a new asynchronous AVG—active fantasy sports—designed to promote physical activity.

**Methods:**

We conducted two pilot studies of an active fantasy sports game designed to promote physical activity. Participants wore a low cost triaxial accelerometer and participated in an online fantasy baseball (Study 1, n=9, 13-weeks) or fantasy basketball (Study 2, n=10, 17-weeks) league. Privileges within the game were made contingent on meeting weekly physical activity goals (eg, averaging 10,000 steps/day).

**Results:**

Across the two studies, the feasibility of integrating physical activity contingent features and privileges into online fantasy sports games was supported. Participants found the active fantasy sports game enjoyable, as or more enjoyable than traditional (sedentary) online fantasy sports (Study 1: *t*_8_=4.43, *P*<.01; Study 2: *t*_9_=2.09, *P*=.07). Participants in Study 1 increased their average steps/day, *t*_8_=2.63, *P*<.05, while participants in Study 2 maintained (ie, did not change) their activity, *t*_9_=1.57, *P*=.15). In postassessment interviews, social support within the game was cited as a key motivating factor for increasing physical activity.

**Conclusions:**

Preliminary evidence supports potential for the active fantasy sports system as a sustainable and scalable intervention for promoting adult physical activity.

## Introduction

### Background

Traditional video games have been sedentary in nature, requiring small hand or thumb movements and very little physical exertion. By contrast, active video games (AVGs) (also known as: exergames, exertainment, or active gaming), link participants’ physical activity to various aspects of video game play, thereby promoting greater physical exertion. In 2011, the American Heart Association published a science panel proceedings report highlighting the promise of AVGs and encouraging further research on their efficacy [1].

Unfortunately, active video games have to this point proven relatively limited in their effectiveness. In particular, empirical tests of active video games that link players’ physical activity with game features in real-time (ie, synchronously) have only minimally and temporarily increased physical activity [2]. Further, the intensity of the exercise achieved while playing many of the most popular synchronous active video games (eg, Wii, Kinect, Move) has fallen below the minimal threshold for moderate intensity physical activity [3]. An additional challenge is economic; thus far, “neither children nor parents . . . have purchased enough healthy videogames to make it profitable” [2].

Theoretical and evidence-based strategies for designing more effective AVGs have been proposed [1,4,5]. Self-determination theory (SDT) is a macro-theory of human motivation that has been applied to identifying factors that might sustain individuals’ motivation in general (eg, in classrooms, the workplace, and in sports) [6], as well as within sedentary video games [5] and AVGs specifically [7,8]. SDT postulates that the more often basic psychological needs for autonomy, competence, and relatedness are satisfied within a game, the more enjoyable and satisfying the experience, and thereby more sustainable the motivation [9]. These three basic psychological needs can be supported in innumerable ways within games. Games can support the need for autonomy by providing participants with opportunities to explore or make choices. When challenges within a game are calibrated to stretch a participant’s ability (ie, by optimal challenge), the need for competence is satisfied. Finally, the need for relatedness is supported when people feel connected to others, which can occur with fictional characters depicted in games, or with real people, as in some multiplayer video games. Research supporting the integration of social support into AVGs has been particularly compelling [10], even with a virtual other or cyber buddy [11].

Baranowski and colleagues [2] also identified evidence-based factors that might improve the efficacy of AVGs. Inclusion of a meaningful and engaging narrative seems to help sustain engagement with AVGs [12]. Additionally, asynchronous (versus synchronous) integration of participant activity with game features may elicit more generalizable and sustainable changes in physical activity. Asynchronous AVGs integrate video game features with activity that takes place when participants are not engaging directly with the gaming interface (eg, through use of an accelerometer that monitors steps over the course of an entire day or week). Participants playing an asynchronous AVG may be sedentary while engaging directly with the gaming interface; the activity occurs asynchronously, (ie, at a different time). While asynchronous AVGs are promising for motivating sustained increases in physical activity, relatively few have been developed (versus synchronous AVGs). Several exceptions include the now defunct Ubifit garden [13], Pokewalker, and Gemini [14] games, as well as smartphone integrated game applications like Nike+’s NikeFuel Missions, and Six to Start’s Zombies Run! series; but to the best of our knowledge, no rigorous empirical tests of these asynchronous AVGs have been published.

Although new AVGs could be developed using these theoretical and empirical strategies, a more economical alternative approach is to integrate physical activity into sedentary video (or online) games that have proven appeal. Currently, one of the most popular and enduring online games among adults in North America are variations of a game platform collectively referred to as *fantasy sports*.

### Online Fantasy Sports as a Platform for Building an Asynchronous Active Video Game

Fantasy sport (also known as rotisserie, roto, or owner simulation) is a game that involves participants selecting professional athletes, and earning points based on the real world performance of those professional athletes. The most common variants of this game involve a group (or league) of participants acting as fantasy team “owners.” Each fantasy team owner drafts and manages a roster of uniquely claimed players over the course of a professional sport season (see Figure 1 in Multimedia Appendix 1); a typical fantasy “league” might include 8 to 12 teams, each managed by an individual team owner (see Figure 2 in Multimedia Appendix 1). Fantasy team owners have the ability to trade, cut, and sign players in a manner analogous to the real owners of professional sport teams (see Figure 3 in Multimedia Appendix 1). Online hosts of fantasy sport games provide automated scoring and opportunities for electronically mediated discussion between league owners, including message boards and direct messaging (see Figure 4 in Multimedia Appendix 1).

For a number of reasons, we hypothesized that online fantasy sports, a traditionally sedentary online game, might represent a gaming platform uniquely well suited to supporting a scalable, sustainable AVG for increasing physical activity. First, traditional (sedentary) fantasy sports are extremely popular (>41 million players in the US and Canada, alone), supporting high potential reach [15]. Second, fantasy sports have established enduring appeal, supporting prolonged engagement; participants play in leagues that last for several months, and often play season-after-season for multiple years within the same league. Popularity and sustained motivation may be related to the ample opportunities for experiencing psychological need satisfaction afforded while playing traditional fantasy sports games. Specifically, this platform provides game players with many options for customizing their teams in ways that can reflect their identities (e.g., selecting athletes for reasons that include their personalities, shared cultural, or regional affiliations), thereby supporting *autonomy need satisfaction*; this platform facilitates finding leagues that include similarly skilled opponents, thereby optimizing challenge, and *competence need satisfaction*; and fantasy sports leagues are social, providing opportunities for interpersonal interaction and *relatedness need satisfaction*.

Third, traditional fantasy sports already involve a form of asynchronous integration between physical activity (that of professional athletes) and game features; as such, fantasy sports games may be especially well suited to asynchronous integration of participants’ physical activity (as recommended by Baranowski et al [2]. Fourth, the basic features of traditional fantasy sports are in the public domain and thus economically and practically amenable to augmentation.

Fifth, although traditional fantasy sports are sedentary, thematically these games involve attending to physical activity data generated by professional athletes. Thus, asking individuals to attend to their own physical activity data is copasetic with traditional fantasy sports in terms of attending to data, and specifically data related to physical activity. Further, we hypothesized that the game’s defining theme of professional sports would have several potential advantages. For example, activating thoughts related to physical activity (regarding professional sports) might prime greater activation of participants’ goals to increase physical activity by virtue of shared semantic and associative networks. Nonconscious priming of goals has previously been shown to increase intensions to pursue those goals [16]. Further, fans of professional sports often identify very strongly with professional sports teams, often fanatically/intensely so, a motivational dynamic that has previously been effectively leveraged for promoting physical activity among overweight and obese male soccer fans in Europe in the context of an intervention delivered in-person [17]. Relatedly, the strong appeal of professional sports, and as such online fantasy sports, to men (approximately 80% of fantasy sports participants are male; Fantasy Sports Trade Association FSTA, 2013), may be especially advantageous in light of evidence that men have traditionally been underrepresented in behavioral health interventions [18], and relatively less willing to engage in health-related online support communities [19].

The primary purpose of this pilot research was to test the feasibility of an active fantasy sports intervention. We tested this in the context of two studies, using two versions that were focused on different sports (baseball and basketball). Feasibility was assessed in terms of participants’ self-reported satisfaction with the game overall and with specific features. An exploratory aim of this research tested whether participants increased their physical activity while playing the game, as objectively assessed by accelerometers.

## Methods

### Study 1: Active Fantasy Baseball

#### Overview

To test the feasibility of the proposed active fantasy sports system, we conducted a 13-week proof-of-concept field study from April to June 2013. The focal sport selected was Major League Baseball, a sport that was integral to the early development of traditional (sedentary) fantasy sports.

#### Recruitment

Participants were recruited using a convenience sampling procedure that involved reaching out to the principle investigator’s online social network. This included inviting individuals with whom the principal investigator had participated in prior traditional fantasy sports leagues. The investigative team considered a number of potential issues related to using this form of convenience sampling (e.g., social desirability bias and experimenter demand characteristics), but opted in favor, given the early formative phase of research and low fidelity execution that could be offered at this stage. No financial incentives or other compensation were offered in exchange for 13-weeks of participation.

Inclusion criteria were highly inclusive; participants had to be over 18 years of age and able to engage in physical activity without significant risk of injury. We intentionally did not exclude individuals who were meeting the recommended amount of physical activity at baseline in order to mirror real-life fantasy sports leagues where players have varying levels of fitness; these inclusion criteria help maximize ecological validity and the potential reach of our intervention.

#### Protocol

After the nature of the study was explained and participants provided their written informed consent, all participants completed a set of preintervention questionnaires. A screening tool (Physical Activity Readiness Questionnaire, PAR-Q) was administered to confirm that the participants were physically prepared to increase their physical activity without significantly risking injury; this measure was designed for adults aged 15-69, and asks about 7 physical activity risk factors (eg, chest pain, dizziness, and joint problems) [20]. All participants passing screening then received an accelerometer to wear for at least 1-week of baseline recording. During the baseline activity recording phase, participants were asked to maintain their activity level. Each participant’s baseline activity was subsequently used to set individually calibrated weekly physical activity goals during the 12-week intervention phase.

Those participants who exceeded the recommended 10,000-steps/day average during the baseline activity recording phase (n=3) were asked to maintain their baseline average each week during the 12-week intervention phase. This policy was instituted as a conservative precautionary measure to ensure the safety of our participants, as well as to purposely deemphasize the value of excessive activity. Those participants who fell short of the recommended 10,000-steps/day average during the baseline activity recording phase were asked to increase their activity by 10% each week, up until their weekly goal became 10,000 steps/day and was capped at that level.

During the 12-week intervention phase, participants competed in a fantasy baseball league hosted by Yahoo. The principle investigator, serving as league commissioner, managed game-related consequences using the league commissioner tools provided by Yahoo (and standard to other major online fantasy sport hosts, including ESPN and CNNSI) once a week. Cumulatively, these managerial tasks performed by the principal investigator required less than 30 minutes each week; these managerial tasks are described in more detail below. Figure 5 in Multimedia Appendix 1 includes screenshots with accompanying explanations of the league commission tools provided by Yahoo. Game-related consequences tied to physical activity during the 12-week intervention phase were two-fold. First, failure to meet a weekly activity goal resulted in the loss of one player (professional athlete) from that participant’s team roster. The player dropped was selected at random, but could not include one of the best (high value) players from a list maintained and updated weekly by Yahoo (ie, the can’t cut list). This made the consequences for failing to meet a weekly goal meaningful, but not extreme. Second, at the end of each week, the league’s waiver wire priority was reset by the principal investigator based on the rank order of participants’ percent of activity goal completed. If and when two or more team owners request to claim the same player who has recently been dropped from another team, the waiver wire priority is used to determine which team owner will be awarded that player. Participants were updated on their physical activity goal progress (and that of others in the league) twice a week via messages posted to the league message board. Participants were able to access their own physical activity data at any time via Fitbit’s website or smartphone application; similarly, the fantasy league itself was available to participants via both Yahoo’s website and smartphone application.

Finally, at the end of the 12-week intervention phase, all participants completed a structured interview with a study staff member by phone or in-person. Participants provided feedback on their experiences using activity-contingent features of the game, and were also given an opportunity to propose new features. Procedures were approved by the Institutional Review Board at the Illinois Institute of Technology IIT.

#### Materials

##### Preintervention Measures

Self-reported demographic information provided at baseline included: age, sex, race, education, height, and weight. Participants also reported their prior experience playing traditional versions of online fantasy sports and self-reported their physical activity over the month prior to enrolling in the study using the Godin Leisure Activity Measure [21]. Intrinsic motivation, specifically enjoyment for exercise in general, was assessed using a 3-item version of the Intrinsic Motivation Index, Cronbach alpha=0.93 [22].

##### Objective Physical Activity

During the 1-week baseline activity recording phase and 12-week intervention phase, physical activity was objectively assessed using a low-cost triaxial accelerometer (a Fitbit Zip). Fitbit’s proprietary algorithms convert raw accelerometer data into estimates of steps taken, calories burned, and distance traveled. For the sake of parsimony, we focused exclusively on steps taken and set weekly goals based on average steps/day. Lee, Kim, and Welk [23] found that the FitBit Zip achieved energy expenditure estimates within 10.1% of criterion values concurrently obtained from a portable metabolic system (ie, Oxycon Mobile).

##### Postintervention Measures

Participants provided quantitative feedback on their experience related to intrinsic motivation for physical activity and for the active fantasy sports game, their attitudes toward different activity-contingent features of the game and new proposed features, and their willingness to play active fantasy sports in the future. Intrinsic motivation (for exercise in general and for the active fantasy sports game in particular) was assessed using separate 3-item versions of the Intrinsic Motivation Index; Cronbach alpha=0.88, and 0.71, respectively [22]. Attitudes toward specific features and willingness to play were assessed using single item Likert-scale questions created for this study.

### Study 2: Active Fantasy Basketball

#### Overview

A second pilot study was run for 17 weeks from November 2013 to February 2014 using a fantasy basketball league (specifically, the National Basketball Association).

#### Recruitment

Study 2 participants were recruited using poster advertisements on a medium size, urban university campus in the Midwestern-region of the United States. Although participants in Study 1 reported a strong preference for knowing everyone in their league cohort in advance, we felt it was important to test the feasibility of the system within a league comprised of strangers-at-baseline. Interested individuals emailed the study’s principle investigator, received a brief overview of the study, and were screened for eligibility.

#### Protocol

After recruitment, the protocol for Study 2 essentially mirrored that for Study 1. Only a few aspects of the protocol were changed. First, the intervention period lasted for 16 weeks (rather than 12). Second, during the intervention period, participants who recorded fewer than 4000 steps in a day were given credit for 4000 steps. This change was made in order to avoid having participants severely punished in the event that they left their accelerometer at home on one or more days and because it was more logistically feasible to execute. Third, participants were offered the opportunity to win small competition-contingent financial incentives based on league performance (1^st^ place was awarded US $150; 2^nd^ place was awarded US $100; 3^rd^ place was awarded US $50, with each subsequent place lowering in increments until the 8^th^ and final place).

## Results

### Study 1: Active Fantasy Baseball

#### Sample Demographics

##### Summary

There were 9 adult males who participated with 100% retention over the 13-week study. Mean age was 34.89 years (SD 2.26, range 32-40). Of the male participants, 8 were White and 1 was Asian. The sample was highly educated, all participants had “some college” or higher. Mean body mass index was 25.20 (SD 1.80, range: 22.38-27.70), which falls slightly above the normal range. The average number of online fantasy sports leagues played prior to the study was 15.70 (SD 19.57, range 0-64). There were 2 participants who had never played traditional fantasy sports before.

##### Enjoyment

Self-reported enjoyment of the active fantasy baseball game was significantly higher than neutral (3) on the 7-point scale, mean 5.59 (SD 1.22), one-sample t_8_=6.36, *P*<.001. Further, participants rated active fantasy sports as significantly more enjoyable compared to traditional fantasy sports, one-sample t_8_=4.43, *P*<.01. Participants self-reported intrinsic motivation for exercising in general did not change significantly from preintervention (mean 4.59, SD 1.52) compared to postintervention (mean 5.15, SD 1.57); paired t_8_=1.72, *P*=.13. Findings are summarized in Table 1.

**Table 1:** Comparison of demographics and outcomes in studies 1 and 2.

Variable	Study 1 (n=9)	Study 2 (n=10)
	Active Fantasy Baseball	Active Fantasy Basketball
	n (%) or mean (SD)	n (%) or mean (SD)
Age in years	34.89 (2.26)	25.50 (3.89)
% male	10 (100%)	9 (90%)
Leagues played in previously / experience	15.70 (19.57)	3.36 (3.96)
BMI	25.20 (1.80)	23.61 (3.18)
Godin activity score at baseline	52.88 (30.36)	40.09 (14.69)
Steps/day at baseline	8678.44 (1784.50)	6961.70 (2137.85)
Steps/day across the intervention phase	11,364.22 (3426.11)	6267.34 (1877.58)
Enjoyment of active fantasy game	5.59 (1.22)	5.23 (1.36)
Number of posts to message board	9.22 (8.57)	1.50 (2.42)
Number of co-participants known well at baseline	4.22 (2.68)	0.60 (0.52)
Preference for knowing co-participants at baseline	2.33 (0.71)	2.10 (1.10)
Preference for everyone being equally active at baseline	0.67 (1.94)	1.00 (2.00)
Ideal impact of physical activity on the game	5.00 (1.23)	5.70 (1.16)
Actual impact of physical activity on the game	3.11 (1.05)	3.70 (1.57)

##### Social Factors

During postintervention interviews, participants frequently cited social aspects of the game as especially motivating and enjoyable. The mean number of posts to the league message board per participant was 9.22 (SD 8.57, range 0-25). On average, they reported knowing 4.22 (SD 2.68) other participants in the league before the start of the season (ie, just over half the other participants in the league). When asked, “How much better or worse would this game have been if you knew everyone in the league very well before starting the season?” (-3=much worse; +3=much better), the mean response was +2.33 (SD 0.71), a strong preference for knowing everyone in the league before the start of the season. Participant mean responses on the social aspects of the game are listed in Table 1.

##### Relative Impact of Fitness

Finally, we asked participants about their preferences with regard to the relative influence of their physical activity versus sedentary strategy on game outcomes. We asked, “If you could choose the degree or amount that team owners’ physical activity impacted the outcomes of an active fantasy sports league, how impactful would you make it?” (1=a very small impact / the least active owner could still win; 7=a very large impact / the least active owners could not win). The mean response was 5.00 (SD 1.23). Next, we asked, “In this pilot study, how much do you think team owners’ physical activity impacted the league outcomes?” (using the identical 7-point scale). The mean response was 3.11 (SD 1.05). The mean difference between these two ratings (mean 1.89) was statistically significant, paired t_8_=3.69, *P*=.006.

##### Physical Activity

Participants’ self-reported and accelerometer-measured physical activity varied at baseline (the mean Godin activity score was 52.88, SD 30.36, range 14-119, and mean steps/day 8678.44, SD 1784.50, range 5913-11,053). Across the 12-week intervention phase, participants averaged over 30% more activity, paired t_8_= 2.63, *P*≤.05 (12-week mean 11,364.22, SD 3426.11, range 4064-15,949).

Given that the 3 participants who were already exceeding the goal of 10,000 steps/day at baseline were asked to maintain their baseline level of activity, we also calculated the mean change in activity for two subgroups, (ie, those given goals to maintain) (n=3, mean change = +2962.67, SD 3136.51) and those given goals to increase activity (n=6, mean change = +2547.33, SD 1357.78); both groups increased their activity.

### Study 2: Active Fantasy Basketball

#### Sample Demographics

##### Summary

A total of 15 applicants responded to recruitment materials expressing interest in the study; 3 did not wish to enroll due to lack of interest. Of the 12 adult participants enrolled in the study, 2 participants dropped out of the study after the first week of the intervention, leaving us with a sample size of 10 (9 males, 1 female). The two participants who dropped out had not played online fantasy sports previously, quickly decided that the game was more complicated than anticipated, and were unwilling or unable to invest time in learning the rules. Mean age was 22.50 (SD 3.89, range 18-30). Of the participants, 4 were White, 4 were Asian, 1 was Black, and 1 was Multiracial. The sample was highly educated; all participants had “some college” or higher. Mean body mass index was 23.61 (SD 3.18, range 19.31-29.18), which is within the normal weight range. The average number of online fantasy sports leagues played in prior to the study was 3.36 (SD 3.96, range 0-9); 6 participants had never played traditional fantasy sports before.

##### Enjoyment

As in Study 1, self-reported enjoyment of the active fantasy sports game itself was significantly higher than neutral (3) on the 7-point scale, mean 5.23 (SD 1.36), one-sample t_9_=5.19, *P*≤001. Furthermore, when asked to compare active fantasy sports relative to traditional (sedentary) fantasy sports in terms of enjoyment, participants rated active fantasy sports as marginally more enjoyable, one-sample t_9_=2.09, *P*=.07. Participants’ self-reported intrinsic motivation for exercising in general did not change significantly from preintervention (mean 4.63, SD 1.64) compared to during the intervention (mean 4.639, SD 2.18).

##### Social Factors

The mean number of posts to the league message board was significantly smaller than in Study 1, mean 1.50 (SD 2.42, range 0-6). On average, they reported knowing 0.60 (SD 0.52) other participants in the league before the start of the season. When asked, “How much better or worse would this game have been if you knew everyone in the league very well before starting the season?” (-3=much worse, +3=much better), the mean response was +2.10 (SD 1.10), a strong preference for knowing other league members. However, when asked: “How much better or worse would this game have been if everyone in the league was roughly as active as you were at baseline?” (-3=much worse; +3=much better), the mean response was 1.00 (SD 2.00), indicating no clear preference.

##### Relative Impact of Fitness

Finally, we asked participants about their preferences with regard to the relative influence of their physical activity versus sedentary strategy on game outcomes. We asked, “If you could choose the degree or amount that team owners’ physical activity impacted the outcomes of an active fantasy sports league, how impactful would you make it?” (1=a very small impact / the least active owner could still win; 7 a very large impact / the lease active owners could not win). The mean response was 5.70 (SD 1.16). Next, we asked, “In this pilot study, how much do you think team owners’ physical activity impacted the league outcomes?” (using the identical 7-point scale). The mean response was 3.70 (SD 1.57). The mean difference between these two ratings (mean 1.89) was statistically significant, paired t_9_=3.00, *P*=.015, indicating that participants wanted physical activity to be more impactful on league outcomes.

##### Physical Activity

Participants’ self-reported and accelerometer-measured physical activity varied at baseline (the mean Godin activity score was 40.09 (SD 14.69) range 14.00-65.50 and mean steps/day 6961.70, SD 2137.85, range 3346-10,473), and during the 16-week intervention phase (mean 6267.34, SD 1877.58, range 4427.97-9607.24). Physical activity did not significantly increase during the intervention phase, paired t_9_=1.57, *P*≤.15.

There was one participant who was already exceeding the goal of 10,000 steps/day at baseline and was asked to maintain his or her baseline level of activity. Change in activity for that participant (n=1, mean change = +865.76) was higher than for those given goals to increase activity (n=9; mean change =-75.31, SD 1481.79).

## Discussion

### Principal Findings

The present investigation demonstrates the feasibility of linking physical activity goals to features and privileges within an extremely popular sedentary video game, online fantasy sports. There were high rates of retention in both studies (zero attrition in Study 1, two dropouts very early in Study 2), and regular posting to the league message board in Study 1. Especially encouraging findings from this study related to participants’ ratings of the degree to which they enjoyed playing active fantasy sports. Across both studies, participants reported enjoying active fantasy sports, and rated it significantly higher than neutral. In Study 1, participants rated active fantasy sports as even more enjoyable than traditional fantasy sports; Study 2 participants rated it as neither more nor less enjoyable than traditional. Study 2 participants had less previous experience with fantasy sports and knew fewer participants in the league, which likely influenced their enjoyment of the league.

Participants in both Studies 1 and 2 reported that knowing other participants in the league at baseline would be highly desirable. As such, we believe interventions of this kind have the best probability of success if groups of friends can be recruited for participation together. These circumstances also more faithfully reflect the typical way that traditional (sedentary) online fantasy sports leagues form.

Although the two pilot studies were not designed or powered to test the system’s potential for increasing physical activity, encouraging findings were observed. The use of accelerometers provides an objective indicator of changes in physical activity, and can be considered a major strength. In Study 1, participants increased their physical activity by over 30% during the active fantasy baseball season compared to baseline. We note that in both studies, participants who were already meeting the activity goal of 10,000 steps/day at baseline were instructed only to maintain their level of activity (3 participants in Study 1, 1 participant in Study 2).

### Limitations

Design limitations include the lack of a control or active comparison condition, and the low fidelity (manual, as opposed to automated) execution of the integration between physical activity data and fantasy sports game features. Consequences were administered manually by the principle investigator using standard tools provided by Yahoo to league commissioners. A more elegant execution of the system could be achieved by leveraging open APIs made available by both accelerometer providers (eg, Fitbit) and fantasy sports league hosts (eg, Yahoo, Espn, or CNNSI). Despite these limitations, the important contribution of swifter, proof-of-concept trials of this kind is being championed in the emerging field of technology-support and mobile health research [24].

The samples in both studies were small (n=9, and n=10), and were not representative of the general population, especially with regard to gender. This limits the generalizability of the findings. However, we note that the sample demographics were relatively consistent with the population of adults who play traditional online fantasy sports (ie, predominately male, Caucasian, and well-educated). We also note that participants’ self-reported height and weight were potentially biased; future studies should include measurements of height and weight taken by research staff members with appropriately calibrated instruments.

### Future Directions

The present research suggests a number of directions for future research. First, leveraging open APIs associated with accelerometer providers and fantasy sport hosting websites will reduce the burden on research staff, allowing researchers to collect data from much larger and more representative samples. Second, it will be important to test the efficacy of the active fantasy sports system for increasing physical activity relative to a usual care or active comparison group (eg, participants assigned to receive an accelerometer for self-monitoring purposes and a group message board only) using a randomized controlled trial. Group-randomization could be employed in order to recruit groups of participants who know each other before enrolling in the study. Third, aspects of the game can potentially be tailored according to the characteristics and preferences of the participants. For example, participants in both studies expressed a desire for their physical activity to have a greater impact on game outcomes. Optimization study designs may help us refine which activity contingent features are maximally effective, test different ways of measuring physical activity (eg, with wearable technology or specialized exercise equipment), and assess different rules for setting activity goals in order to maximize both optimal challenge and perceived fairness. Prior research also shows that levels of competitiveness may influence enjoyment of games and thus may have implications for intrinsic motivation [25,26]; future research may tailor game features or match players based on competitiveness. Fourth, given the preliminary evidence that social factors helped motivated participation and intervention success, future research should investigate this issue further (eg, qualitative and quantitative analysis of message board usage). Fifth, future research may expand the variety of target sports (eg, football, soccer, hockey, golf, rugby, cricket, etc), thereby potentially increasing reach.

### Potential Public Health Impact

As noted in the introduction, Baranowski and others [2] have argued that the public health impact of previous AVGs has been limited by low uptake and high cost. As such, we think it is useful to highlight a number of unique strengths associated with active fantasy sports interventions that support potential reach and cost-effectiveness.

Importantly, we believe the established massive popularity of traditional (sedentary) fantasy sports provides a powerful dissemination channel. In 2014, over 41 million adults in the United States (US) and Canada alone played online fantasy sports [15]. For reference, in 2014, the entire population of Canada was roughly 34 million. If even a fraction of the traditional fantasy sports audience could be persuaded to try active fantasy sports, this would represent significantly greater reach than previous AVGs. Further, while most AVGs have not made social features integral or required, preliminary evidence suggested that social aspects of the active fantasy sports intervention were important. As such, active fantasy sport interventions may be especially well suited to implementation by organizations interested in promoting physical activity (eg, schools, or employer sponsored wellness programs). Active fantasy sports leagues sponsored by organizations could significantly magnify reach and, by leveraging existing social ties, may also improve efficacy.

Further supporting the potential reach of active fantasy sport type interventions are a number of factors limiting costs associated with software development, labor, and equipment. As noted earlier, the game platform that active fantasy sports interventions are built on (traditional fantasy sports) is in the public domain and supported by numerous companies (“host” websites, including Yahoo, ESPN, and CNNSI). By taking advantage of existing, sophisticated game platform infrastructure provided by numerous hosts, developers achieve significant cost reductions relative to building a comparable AVG de novo. The major hosts of traditional online fantasy sports offer open API access to external application developers specifically to encourage innovation of this kind. Furthermore, established hosts currently offer consumers access to traditional fantasy sports games without charging fees (major hosts typically use an advertising-based revenue model), minimizing an important barrier for low-income consumers. At present, the overhead and labor costs associated with administrating an active fantasy sports intervention are already very low; the intervention was administered remotely (as opposed to in-person) and involved minimal administrative effort (<1 hour per week). By leveraging device and host APIs, future iterations of this intervention may further automate oversight and reduce costs, especially relative to gold-standard interventions such as individual professional coaching delivered in-person. A third factor related to cost concerns the affordability of equipment used for objectively measuring physical activity. The present versions of the active fantasy sports intervention employed a low cost accelerometer (retail price US $60) that was provided to participants. However, the cost of dedicated wearable accelerometers is rapidly falling; similar models are currently available for as low as US $12 per unit [27]. Smartphones, which typically include sophisticated built-in accelerometers, are increasingly ubiquitous, reducing the cost of activity tracking equipment further still. The cost of more sophisticated activity tracking devices (eg, activity trackers that incorporate heart rate sensors, global positioning system, and accelerometry, including Apple’s iWatch, Fitbit’s Surge, and Microsoft’s Band) should similarly fall if the proliferation of wearable activity tracking technology continues.

### Conclusions

To summarize, this study provides strong support for the feasibility of integrating physical activity contingent features and privileges into an online fantasy sports game platform. Participants found this asynchronous active video game system enjoyable, as or more enjoyable than traditional (sedentary) online fantasy sports. Social support within the game was cited as an important factor for both enjoyment and increasing physical activity. Future research is needed to test the efficacy of this system using a randomized controlled trial and optimization-type designs, and with a larger, more diverse sample of participants. The scalability and reach of active fantasy sport interventions represents a significant strength.

## Abbreviations

AVG: active video game
SDT: self-determination theory
FSTA: Fantasy Sports Trade Association
PAR-Q: Physical Activity Readiness Questionnaire
IIT: Illinois Institute of Technology

## Multimedia Appendix 1

Screenshots of a fantasy sports team roster, a league comprised of multiple fantasy sports teams, a fantasy team owner tool for offering a player trade to another fantasy team owner, a fantasy sports league message board, and fantasy sports league commissioner tools.
