# Supplementary material for: Active Fantasy Sports: Rationale and Feasibility of Leveraging Online Fantasy Sports to Promote Physical Activity
Source: JMIR Serious Games. 2014 Nov 25;2(2):e13. doi: 10.2196/games.3691 (PMC4307829; doi:10.2196/games.3691)
Supplement: Supplementary file 1 [file games_v2i2e13_app1.pdf]

Multimedia Appendix

Figure 1: An Example of a Fantasy Sports Team Roster

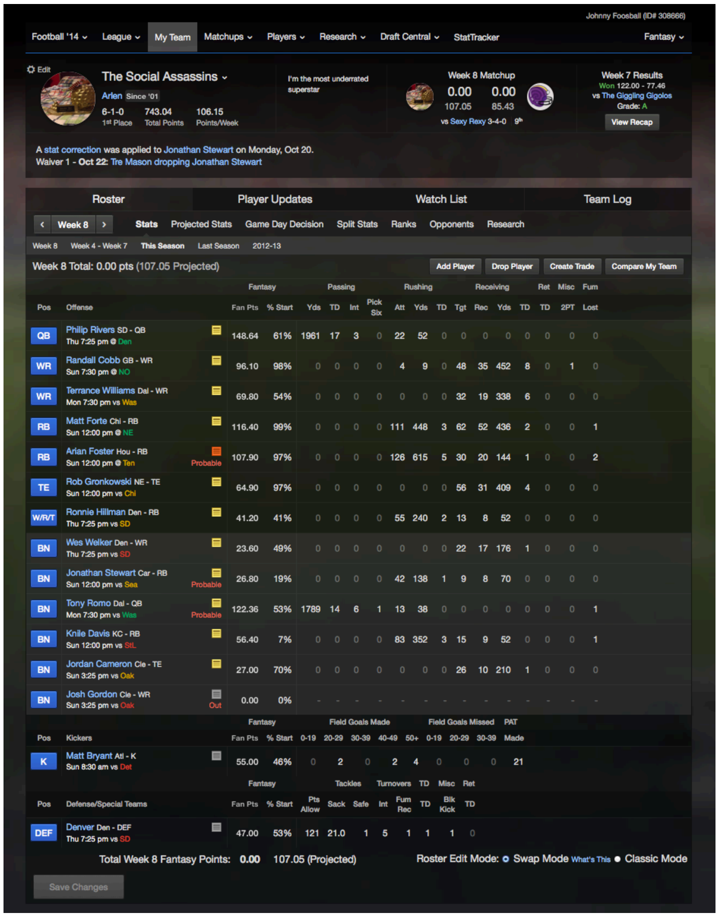

Figure 2: A League Comprised of Multiple Fantasy Sports Teams

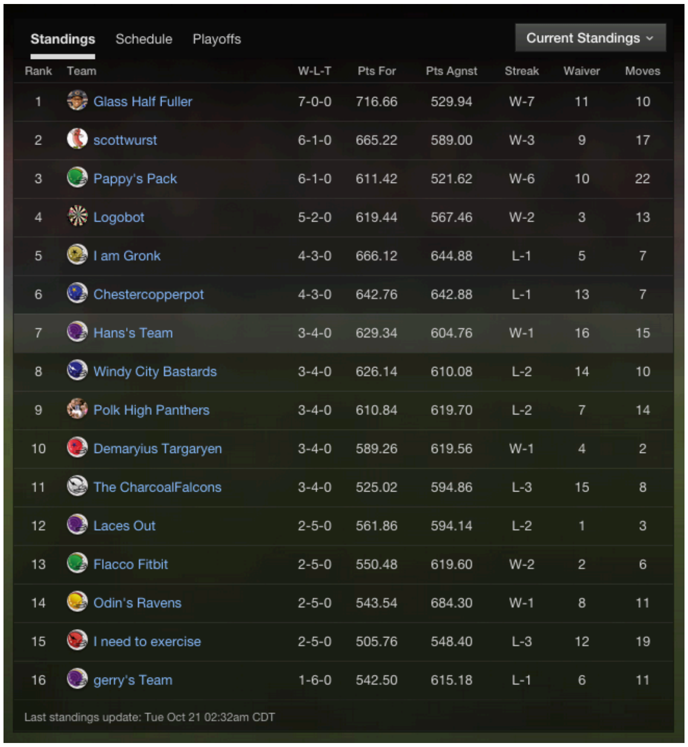

Figure 3: A Fantasy Team Owner Tool for Offering a Player Trade to Another Fantasy Team Owner

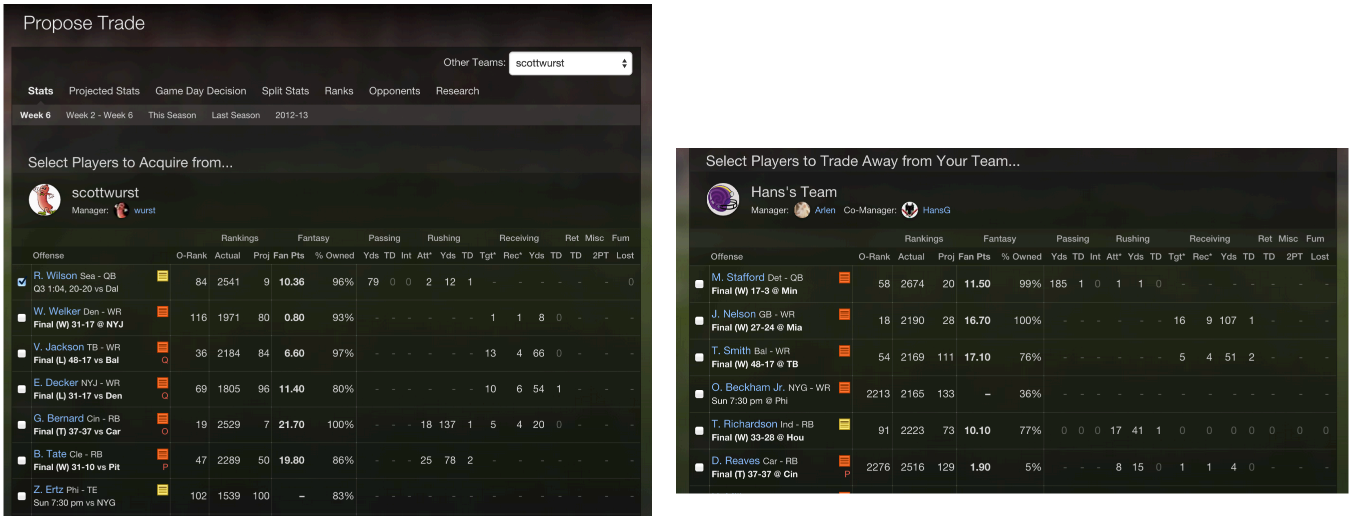

Figure 4: A Fantasy Sports League Message Board

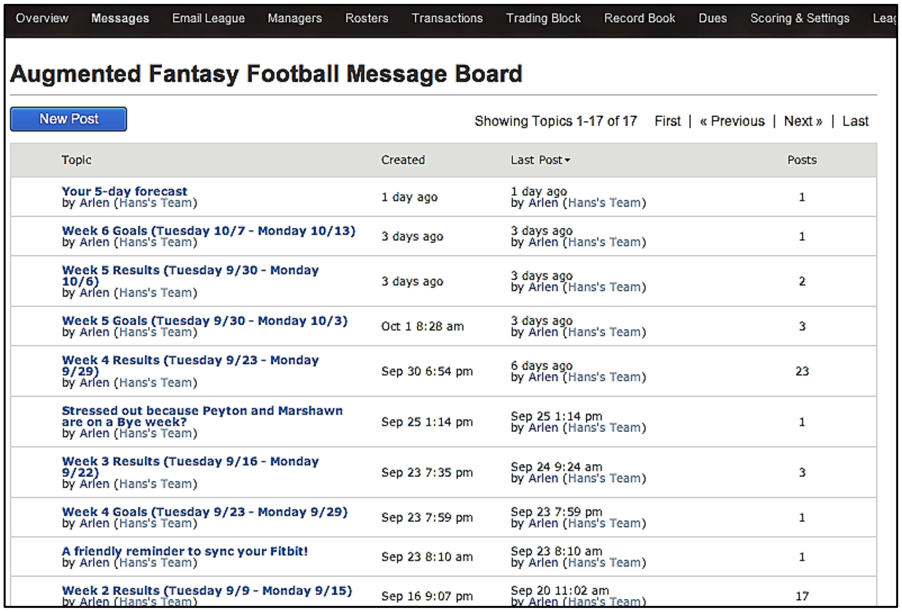

Overview | Messages | Email League | Managers | Rosters | Transactions | Trading Block | Record Book | Dues | Scoring & Settings | Leag

Augmented Fantasy Football Message Board

New Post

Showing Topics 1-17 of 17 First | « Previous | Next » | Last

Topic	Created	Last Post	Posts
**Your 5-day forecast** by Arlen (Hans's Team)	1 day ago	1 day ago by Arlen (Hans's Team)	1
**Week 6 Goals (Tuesday 10/7 - Monday 10/13)** by Arlen (Hans's Team)	3 days ago	3 days ago by Arlen (Hans's Team)	1
**Week 5 Results (Tuesday 9/30 - Monday 10/6)** by Arlen (Hans's Team)	3 days ago	3 days ago by Arlen (Hans's Team)	2
**Week 5 Goals (Tuesday 9/30 - Monday 10/3)** by Arlen (Hans's Team)	Oct 1 8:28 am	3 days ago by Arlen (Hans's Team)	3
**Week 4 Results (Tuesday 9/23 - Monday 9/29)** by Arlen (Hans's Team)	Sep 30 6:54 pm	6 days ago by Arlen (Hans's Team)	23
**Stressed out because Peyton and Marshawn are on a Bye week?** by Arlen (Hans's Team)	Sep 25 1:14 pm	Sep 25 1:14 pm by Arlen (Hans's Team)	1
**Week 3 Results (Tuesday 9/16 - Monday 9/22)** by Arlen (Hans's Team)	Sep 23 7:35 pm	Sep 24 9:24 am by Arlen (Hans's Team)	3
**Week 4 Goals (Tuesday 9/23 - Monday 9/29)** by Arlen (Hans's Team)	Sep 23 7:59 pm	Sep 23 7:59 pm by Arlen (Hans's Team)	1
**A friendly reminder to sync your Fitbit!** by Arlen (Hans's Team)	Sep 23 8:10 am	Sep 23 8:10 am by Arlen (Hans's Team)	1
**Week 2 Results (Tuesday 9/9 - Monday 9/15)** by Arlen (Hans's Team)	Sep 16 9:07 pm	Sep 20 11:02 am by Arlen (Hans's Team)	17

Figure 5: Fantasy Sports League Commissioner Tools

Tool allowing League Commissioner to drop players when team

Tool allowing League Commissioner to adjust waiver wire priority based on % of weekly activity goals completed

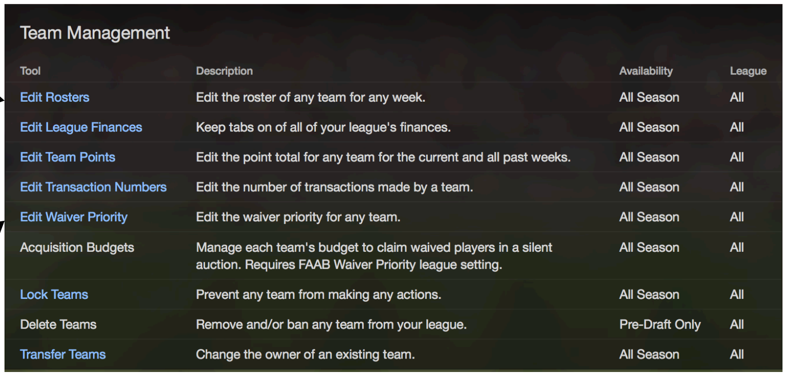

Team Management

Tool	Description	Availability	League
Edit Rosters	Edit the roster of any team for any week.	All Season	All
Edit League Finances	Keep tabs on of all of your league's finances.	All Season	All
Edit Team Points	Edit the point total for any team for the current and all past weeks.	All Season	All
Edit Transaction Numbers	Edit the number of transactions made by a team.	All Season	All
Edit Waiver Priority	Edit the waiver priority for any team.	All Season	All
Acquisition Budgets	Manage each team's budget to claim waived players in a silent auction. Requires FAAB Waiver Priority league setting.	All Season	All
Lock Teams	Prevent any team from making any actions.	All Season	All
Delete Teams	Remove and/or ban any team from your league.	Pre-Draft Only	All
Transfer Teams	Change the owner of an existing team.	All Season	All

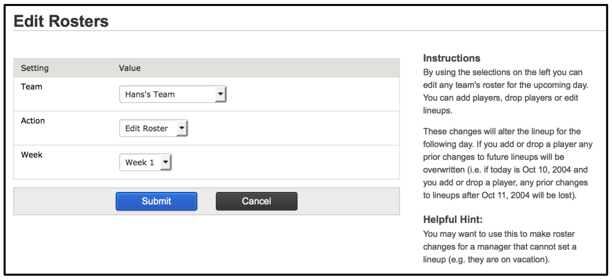

Edit Rosters

Setting	Value
Team	Hans's Team
Action	Edit Roster
Week	Week 1

Submit Cancel

Instructions
By using the selections on the left you can edit any team's roster for the upcoming day. You can add players, drop players or edit lineups.

These changes will alter the lineup for the following day. If you add or drop a player any prior changes to future lineups will be overwritten (i.e. if today is Oct 10, 2004 and you add or drop a player, any prior changes to lineups after Oct 11, 2004 will be lost).

Helpful Hint:
You may want to use this to make roster changes for a manager that cannot set a lineup (e.g. they are on vacation).

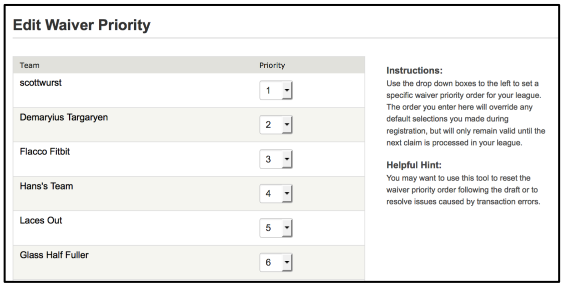

Edit Waiver Priority

Team	Priority
scottwurst	1
Demaryius Targaryen	2
Flacco Fitbit	3
Hans's Team	4
Laces Out	5
Glass Half Fuller	6

Instructions:
Use the drop down boxes to the left to set a specific waiver priority order for your league. The order you enter here will override any default selections you made during registration, but will only remain valid until the next claim is processed in your league.

Helpful Hint:
You may want to use this tool to reset the waiver priority order following the draft or to resolve issues caused by transaction errors.
